# Breast cancer incidence and menopausal hormone therapy in Norway from 2004 to 2009: a register-based cohort study

**DOI:** 10.1002/cam4.474

**Published:** 2015-05-20

**Authors:** Pål Suhrke, Per-Henrik Zahl

**Affiliations:** 1 Department of Pathology, Oslo University HospitalPO Box 4950 Nydalen, N-0424, Oslo, Norway; 2 Department of Pathology, Vestfold Hospital TrustPO Box 2168, N-3103, Tønsberg, Norway; 3 Norwegian Institute of Public HealthPO Box 4404 Nydalen, N-0403, Oslo, Norway

**Keywords:** Breast cancer, hormone therapy, mammography and invasive lobular carcinoma

## Abstract

In Norway, the breast cancer incidence increased by 50% in the 1990s, during a period with initiation of mammography screening as well as a fourfold increase in use of menopausal hormone therapy (HT). After 2002, the HT use has dropped substantially; however, the breast cancer incidence has declined only marginally. How much mammography screening contributed to the breast cancer incidence increase in the 1990s compared with HT use and specifically different types of HT use, has thus been discussed. Whether HT affects the incidence of subtypes of breast cancer differently has also been questioned. We have linked individual data from several national registries from 2004 to 2009 on 449,717 women aged 50–65 years. 4597 cases of invasive cancer and 681 cases of ductal carcinoma in situ (DCIS) were included in the analysis. We used Cox regression to estimate hazard ratio (HR) as a measure of the relative risk of breast cancer associated with use of HT. The HRs associated with prescriptions of HT for more than 1 year were 2.06 (1.90–2.24) for estrogen and progesterone combinations, 1.03 (0.85–1.25) for systemic estrogens, and 1.23 (1.01–1.51) for tibolone. Invasive lobular carcinoma was more strongly associated with use of estrogen and progesterone combinations, HR = 3.10 (2.51–3.81), than nonlobular carcinoma, HR = 1.94 (1.78–2.12). The corresponding value for DCIS was 1.61 (1.28–2.02). We estimated the population attributable fraction to 8.2%, corresponding to 90 breast cancer cases in 2006 indicating that HT use still caused a major number of breast cancer cases.

## Introduction

The breast cancer incidence increased rapidly during the 1990’s 1, and several observational studies linked the increase to widespread use of menopausal hormone therapy (HT) 2–7. After the publication of the randomized controlled Women’s Health Initiative trial in 2002 8 and the large observational Million Women Study in 2003 9, the use of HT has dropped substantially in Norway 10–12. Norwegian observational studies have reported 58% 2 and 110% 3 increased risk of having breast cancer diagnosed for HT users. In Norway, the breast cancer incidence increased by 50% in the 1990s 13, during a period with a fourfold increase in HT use; however, the breast cancer incidence declined only marginally after 2002 10–12. The Norwegian breast cancer screening program (NBCSP) started in 1996 and covered whole of Norway from 2004 14. How much mammography screening contributed to the breast cancer incidence increase in the 1990s compared with HT use has thus been questioned 10,12,15.

Several studies have shown that HT use affects breast cancer risk differently for different histological subtypes, and in particular increases the risk of the second most common subtype of breast cancer, invasive lobular carcinoma 16–19. Whether HT affects the incidence of ductal carcinoma in situ (DCIS), a possible precursor lesion of invasive breast cancer, has been questioned 20.

Here, we have linked individual data from several high-quality Norwegian registries and studied how breast cancer incidence is associated with both different type of HT use and duration of use. The study period begins in 2004, since individual data on prescription of HT are not available before 2004 and the NBCSP covered all Norwegian counties from 2004. We also present data stratified on histological subtypes.

## Material and Methods

### Data

As part of the evaluation of the NBCSP, funded by the Research Council of Norway, we received anonymized individual data from the nationwide Norwegian Cancer Registry, Statistics Norway, the Norwegian Prescription Database, and the Medical Birth Registry of Norway. We included all Norwegian women aged 50–65 years in 2006 in our analysis. Data from the Cancer Registry cover the period until December 31, 2009. All data files included a unique number for each individual woman which enabled us to merge the files. From the Cancer Registry, we used two different files. One consisted of information on mammography screening activity, including scheduled dates and whether or not each woman underwent screening. The second file from the Cancer Registry included information on all cases of breast cancer and DCIS, including detection date, histological tumor type, and detection mode (whether a cancer is detected through screening, detected between two screening rounds [interval cancer], detected in invited women who did not attend screening or detected in women who are not yet invited to screening). Data from Statistics Norway included information on death date, causes of death, and date of emigration. From the Medical Birth Registry, we received information on number of births. Data from the Prescription Database included information on all women who had prescriptions of estrogen preparation or combined estrogen progesterone preparations from 2004, including date of prescription, the name of the drug, the ATC-group, and the defined daily dose (DDD) prescribed (http://www.whocc.no/atc_ddd_methodology/purpose_of_the_atc_ddd_system/). DDD is an estimate on the average maintenance dose of a drug used per day (365 DDD correspond to 1 year use).

### Statistical analysis

We used Cox regression to estimate hazard ratio (HR) as a measure of the relative risk of breast cancer associated with use of HT. Based on ATC codes and sales names, we stratified the HT into four different groups: estrogen and progesterone combinations (G03F), tibolone (G03CX), vaginal estrogens (estriol [G03CA04] and low dose vaginal estradiol [G03CA03]) and finally systemic estrogens without progesterone (G03C, except tibolone and low dose vaginal estrogens). Based on the number of prescribed DDD in 2004 and 2005, the women who were 50–65 years old in 2006 (born 1941–1956) were stratified into four different groups: women with no prescriptions, prescriptions of 1–180 DDD, 181–365 DDD, or more than 365 DDD. The event was defined as the time from January 1, 2006 to either an invasive breast cancer diagnosis or to a DCIS diagnosis. The follow-up period ended on 31.12.2009. We estimated HRs with 95% confidence intervals and adjusted for available risk factors such as age, number of childbirths, and whether or not the woman attended the Norwegian Breast Screening Program between 2004 and 2009. We excluded 84 cases of invasive breast cancer and four cases of DCIS from the analysis because they were detected before the woman got an invitation to screening. We did separate analysis for DCIS and invasive breast cancer and also stratified on histological tumor type, invasive lobular carcinoma, or nonlobular invasive carcinoma.

The population attributable fraction (PAF) is a measurement which describes the proportion of avoidable breast cancer cases if the HT use was eliminated. The PAF was calculated as *P*(HR − 1)/(1 + *P*(HR − 1)), where *P* is the proportion of the population using HT, and HR is the hazard rate for invasive breast cancer.

## Results

The study population includes a total number of 449,717 women aged 50–65 years at the beginning of the study period in 2006. In the analysis, we included 4597 cases of invasive cancer and 681 cases of DCIS diagnosed in 2006–2009. By the end of the study period, 187 of these women had died of breast cancer. Eighty-three percent of the population attended one or more of the three screening rounds in 2004–2009. HT use is described in Table1. In 2004–2005, 26.5% of the population had one or more prescriptions of HT. For estrogen and progesterone combinations, 14% of the population had one or more prescriptions, while 8.4% had prescriptions of more than 365 DDD.

**Table 1:** The number and percentages in parentheses of women aged 50–65 years in 2006 with prescriptions of different types of HT in DDD in 2004 and 2005

Prescription in DDD	Estrogen and progesterone combinations	Systemic estrogens	Tibolone	Low dose vaginal estrogens
0	386,657 (86.0)	430,782 (96.8)	433,996 (96.5)	416,364 (92.6)
1–180	13,653 (3.0)	4583 (1.0)	4981 (1.1)	29,565 (6.6)
181–365	11,602 (2.6)	4428 (1.0)	3185 (0.7)	2935 (0.6)
>365	37,805 (8.4)	9924 (2.2)	7555 (1.7)	853 (0.2)

Table2 shows HRs of invasive breast cancer and DCIS associated with recorded risk factors; age, the number of childbirths, and whether or not the woman attends the mammography screening program.

**Table 2:** Hazard ratio of invasive breast cancer and DCIS associated with available risk factors; age, the number of childbirths, whether or not the woman attends the mammography screening program

Risk factor	Invasive breast cancer		Ductal carcinoma in situ	
	Number	HR with 95% CI	Number	HR with 95% CI
Age in 2006				
50–53	1039		188	
54–57	1101	1.14 (1.04–1.24)	144	0.93 (0.75–1.16)
58–61	1313	1.34 (1.23–1.45)	198	0.75 (0.60–0.95)
62–65	1144	1.53 (1.40–1.67)	151	1.00 (0.81–1.24)
Childbirth				
0	836		118	
1–2	2415	0.95 (0.87–1.03)	354	1.31 (1.04–1.65)
>3	1346	0.75 (0.68–0.82)	209	1.20 (1.01–1.42)
Attending the screening program in 2004–2009				
No	623		36	
Yes	3890	1.15 (1.05–1.24)	640	3.32 (2.37–4.65)

The HRs of invasive breast cancer associated with different types of HT prescriptions and different duration of HT use in 2004–2005 are presented in Table3. For estrogen and progesterone combinations, the adjusted HR associated with prescriptions of more than 365 DDD, corresponding to more than 1 year of use, is 2.06 (1.90–2.24). More short-term users, between half a year and 1 year of use, have a slightly increased risk, HR is 1.24 (1.04–1.47). For women with less than half a year of use of estrogen and progesterone combinations, the breast cancer risk is not increased. For users of systemic estrogens, the breast cancer risk is not increased, independent of the duration of use. Tibolone users have a slightly increased risk for breast cancer, HR is 1.23 (1.01–1.51) for users more than 1 year.

**Table 3:** The HR of invasive breast cancer associated with different type of HT prescription and different duration of use in 2004–2005

Prescription in DDD	Estrogen and progesterone combinations		Systemic estrogens		Tibolone		Vaginal estrogens	
	Number	HR	Number	HR	Number	HR	Number	HR
0	3584	1	4398	1	4390	1	4265	1
1–180	134	1.07 (0.90–1.27	41	0.86 (0.63–1.17)	66	1.32 (1.03–1.68)	290	0.84 (0.74–0.95)
181–365	135	1.24 (1.04–1.47)	52	1.12 (0.85–1.48)	41	1.16 (0.85–1.60)	35	0.93 (0.66–1.30)
>365	744	2.06 (1.90–2.24)	106	1.03 (0.85–1.25)	100	1.23 (1.01–1.51)	7	0.65 (0.31–1.37)

All values are adjusted for age, number of child births, whether or not the women attended the screening program in 2004–2009 and whether or not a nonuser in 2004–2005 started with HT use in 2006–2009.

Table4 shows the risk of DCIS, invasive lobular carcinoma, and invasive nonlobular carcinoma associated with different duration of use of estrogen and progesterone combinations. For women with prescriptions of more than 365 DDD, corresponding to use for more than 1 year, the HR for lobular carcinoma is 3.10 (2.51–3.81) and 1.94 (1.78–2.12) for nonlobular carcinoma. The corresponding value for DCIS is 1.61 (1.28–2.02). For short-term users, less than 1 year, only the nonlobular carcinomas have a slightly increases risk.

**Table 4:** The HR for invasive lobular carcinoma, invasive nonlobular carcinoma and DCIS associated with different duration of use of estrogen and progesterone combinations in 2004–2005

Prescription in DDD	Invasive lobular carcinoma		Invasive nonlobular carcinoma		Ductal carcinoma in situ	
	Number	HR	Number	HR	Number	HR
0	391	1	3193	1	541	1
1–180	16	1.17 (0.71–1.93)	118	1.05 (0.88–1.27)	26	1.26 (0.84–1.89)
180–365	12	1.01 (0.57–1.80)	123	1.26 (1.05–1.51)	23	1.34 (0.88–2.05)
>365	120	3.10 (2.51–3.81)	624	1.94 (1.78–2.12)	91	1.61 (1.28–2.02)

All values are adjusted for age, number of child births, whether or not the women attended the screening program in 2004–2009 and whether or not a nonuser in 2004–2005 started with HT use in 2006–2009.

Based on the calculated HR of 2.06 in table3 for long-term users of estrogen and progesterone combination and the proportion of long-term users of 8.4%, we estimated the PAF to 8.2%, corresponding to around 90 breast cancer cases in 2006.

## Discussion

In this study, we observe a 106% increased risk of breast cancer associated with long-term prescription (>1 year) of estrogen and progesterone combinations. The breast cancer risk is not increased by short term prescription (<½ year). For tibolone, we found a 23% increase in the breast cancer risk for long-term prescription. Our estimates correspond with the estimates in the Million Women Study 9 for tibolone and for estrogen and progesterone combinations, but not for estrogen only HT, since we did not find any association between the prescription of estrogen-only HT and breast cancer risk. The Million Women Study 9 observed a 30% increased risk, while another study from Norway found an 80% increased risk for estrogen only HT users 3. A second Norwegian study did not stratify on the type of HT use, and the risk of estrogen–progesterone combinations could not be separated from estrogen only 2. The PAF was estimated to 8.2% in the age group 50–65 years in 2006, which suggests that even after the decline in HT use after year 2002, 90 cases of breast cancer were still caused by HT use in 2006. Jørgensen and Gøtzsche have estimated 52% overdiagnosis of breast cancer in populations offered organized mammography screening 1. In 2006, there were 1142 cases of invasive breast cancer in women aged 50–65 years in Norway, corresponding to almost 400 overdiagnosed women, suggesting that there were four times more overdiagnosed women than breast cancer cases caused by HT use in 2006. These results combined with only a marginally decline in breast cancer in Norway after 2002, support the theory that breast cancer increase in Norway in the 1990-ties was mainly caused by mammography screening and overdiagnosis.

As already published by several authors 10,16–19, we observe that the risk of the second most common type of breast cancer, invasive lobular carcinoma is more strongly associated with HT use than the risk of other nonlobular invasive subtypes; HR = 3.10 (2.51–3.81) versus 1.94 (1.78–2.12). For DCIS, published data have varied. Data from the WHI trial showed a 23%, nonsignificant increased DCIS risk for users of estrogen and progesterone combinations compared with placebo, while observational data from the same study showed a 65% increased risk for users compared with nonusers 20. Our data give support to these data and suggests that the risk of DCIS is associated with long term use of estrogen and progesterone combinations; HR = 1.61 (1.28–2.02).

We also tried to calculate how breast cancer death was affected by HT use, but since there were only 187 breast cancer deaths in the follow-up period, the data were not able to answer this question.

A strength of this study is the prospective study design: First, we have used individual data from national registries. Second, data on HT use are not affected by the possibility of recall bias as when using questionnaires. Third, we have also included data on whether or not the women attend the Norwegian Breast Screening Program. Due to overdiagnosis, screening increases the risk of both invasive breast cancer and DCIS 1. If HT users attended the screening program differently from non HT users, adjusting for mammography activity would be important. We observe a HR of 1.15 for invasive breast cancer and 3.32 for DCIS for attendees versus nonattendees in 2004–2009.

There are several weaknesses in the study: First, a lack of information on other possible risk factors as menopausal status, age at menarche, family history of breast cancer, and educational level. In particular, we do not have data on attendance to private mammography and cannot adjust well for the most important confounder—mammography screening. The 15% difference in risk of a breast cancer diagnosis between those attending the screening program and those not attending indicates a high level of opportunistic screening. It is generally accepted that initiation of mammography screening increases the incidence of breast cancer by 30–50% 1. An interaction is also possible, that HT only increases the risk for overdiagnosed mammography-detected tumors, with little effect on the number of fatal breast cancers. This study cannot answer how breast cancer mortality is affected by HT use.

Second, the data on drug prescription began in 2004. This means that women defined as long-term users in our analysis, could have been nonusers before 2004 or could have been users for several years. We are not able to separate these women. Similarly women defined as nonusers in 2004–2005 could in fact be past or never users. The Million Women study shows that women who had ceased HT use the previous year had a slight increased breast cancer risk of 14%, although the breast cancer risk was not increased for all past users 9. Follow-up of the WHI trial shows a 27% nonsignificant increased risk of breast cancer for women in the intervention group compared with the placebo group in the first 3 years after intervention was stopped 21. Since breast cancer cases in our analysis are counted from 2006, the nonusers have had no prescription of HT at least 2 years preceding a breast cancer diagnosis. Consequently, our calculations should not be strongly influenced by lacking possibility to separate past from never users.

Third, the information on HT use is based on prescriptions. We do not have information on compliance, but we would assume that women who have more than one prescription with many DDD prescribed, do use the drug more regularly than those having only one prescription with a lower DDD prescribed.

In conclusion, we observe a 106% increased risk of breast cancer associated with long-term prescription (>1 year) of estrogen and progesterone combinations, but we did not find any association between prescription of estrogen-only HT and breast cancer risk. The second most common subtype of breast cancer, invasive lobular carcinoma was more strongly associated with HT use than other subtypes. The risk of the possible precursor lesion, DCIS, was also increased for HT users. The PAF was estimated to 8.2%, corresponding to 90 breast cancer cases in 2006, indicating that even after substantial drop in HT use, still a major number of breast cancer cases were caused by HT-use.
